# Advances in Therapeutic Endoscopy

**DOI:** 10.1155/2019/6253837

**Published:** 2019-03-12

**Authors:** Sooraj Tejaswi, Mohit Girotra, Júlio P. Lima, James H. Tabibian

**Affiliations:** ^1^Division of Gastroenterology & Hepatology, University of California, Davis, Sacramento, California, USA; ^2^University of Miami Miller School of Medicine, Miami, Florida, USA; ^3^Universidade Federal de Ciências da Saúde de Porto Alegre, Porto Alegre, Brazil; ^4^Division of Gastroenterology, Department of Medicine, Olive View-UCLA Medical Center, Sylmar, California, USA; ^5^David Geffen School of Medicine, Los Angeles, California, USA

Advanced endoscopic techniques comprise an exciting and ever-evolving field in medicine, enabling minimally invasive interventions for the management of numerous benign and malignant diseases of the GI tract that historically were possible only with major surgery. This special issue covers many articles from across the globe, examining the role of such techniques in the management of pancreaticobiliary, foregut, and other diseases.

R. Berry et al. discuss the etiology, diagnosis, and management of hemobilia, a potentially life-threatening condition whose incidence has increased in light of the growing frequency of hepatopancreatobiliary procedures, including interventional radiologic and advanced endoscopic. The authors focus on the management of hemobilia from the perspective of an advanced endoscopist, while also providing an overview of the condition and the spectrum of available diagnostic and therapeutic modalities.

Several articles addressed the important topic of pancreatitis, from prevention to management of its sequelae. With regard to the former, administration of rectal indomethacin in the absence of contraindication has become a standard practice to decrease the risk of post-ERCP pancreatitis, especially for patients deemed to be at high risk. In the systematic review and meta-analysis by X. He et al., the authors show that rectal indomethacin is protective not only in high-risk patients, but also in those deemed to be average-risk patients. Interestingly, they also found that pre-ERCP administration of indomethacin was more protective. Studies such as these may help endoscopists who are undecided about the utility of rectal indomethacin in average-risk cases. A. Garber et al. review the mechanisms and management of acute pancreatitis, a major cause of morbidity and hospital admission. They discuss early supportive care, with an emphasis on early and adequate fluid resuscitation and nutrition and on endoscopic management of late complications. This is a timely review that reinforces the American Gastroenterology Association's 2018 guideline on the management of acute pancreatitis. Complementing this article, M. Jagielski et al. describe their experience with different techniques of endoscopic drainage of walled-off pancreatic necrosis (WOPN) and how the different methods have evolved over time.

Management of esophageal disorders, both benign and malignant, has become a staple of endoscopic practice and is discussed in this special issue. For example, Y.-W. Zhang et al. present the findings of a meta-analysis of randomized controlled trials (RCTs) which demonstrates the benefit of intralesional triamcinolone injection after endoscopic dilation in the management of benign esophageal strictures. Complementing this article and on the topic of postesophagectomy anastomotic leakage (a significant cause of mortality and morbidity), S. M. Noh et al. describe how endoscopic vacuum-assisted closure (E-VAC) can be an effective nonsurgical management technique in select cases.

Endoscopic submucosal dissection (ESD) is the topic of four articles included in this special issue, including two pertaining to esophageal ESD. P. Shi and X. Ding provide a succinct review of the various approaches, including pharmacological treatments, esophageal stents, and tissue engineering/cell therapies, to prevent esophageal stricture formation after ESD. As an offshoot of (autologous) cell therapies, M. Uesato et al. describe the “log bridge” method of maximum mucosal preservation in near circumferential esophageal ESD cases; the authors found that this method was associated with quicker ESD site healing and a trend toward less esophageal stricture formation (a trend toward a lower need for subsequent endoscopic balloon dilation). Of the remaining two ESD-related articles, X. Feng et al. examined the efficacy and safety of endoscopic submucosal *tunnel* dissection (ESTD) for the resection of large (mean size 4.6 cm) superficial gastric lesions and compared it to a traditional ESD approach; the authors found excellent results with both, though ESTD was found to have the advantage of shorter resection (i.e., procedure) times. D. Kikuchi et al. investigated whether the treatment of an ESD site with autologous fibrin glue (prepared using autologous blood) alone or with polyglycolic acid (PGA) sheets could decrease the risk of delayed bleeding in patients receiving antithrombotic therapy; of the 20 patients included, none had delayed bleeding. These early results are promising and may suggest the need for a randomized study.

J. W. Choe et al. conducted a RCT comparing the conventionally used uncovered self-expanding metallic stent (SEMS) to a shape-modified partially covered SEMS for the management of gastric outlet obstruction (GOO). The study did not find a significant difference in patency or distal migration; thus, the appropriate stent shape and design (uncovered versus partially covered) for the management of GOO remains an unresolved question and is likely to be the subject of future studies, though it is probable that no single stent will be superior in all clinicoanatomical scenarios.

Finally, Y. Jiang et al. report on the comparable efficacy of cyanoacrylate injection vs. through-the-scope (TTS) clip placement for the management of a bleeding duodenal Dieulafoy lesion. The authors found both techniques to be safe and effective, suggesting that cyanoacrylate glue may be an additional tool for the endoscopist in addition to the more commonly used TTS clips.

We hope that the articles in this special issue will make for useful reading for an audience interested in advanced endoscopy and in the latest advances in endoscopic management of pancreaticobiliary and foregut diseases.

